# Potential application of cell reprogramming techniques for cancer research

**DOI:** 10.1007/s00018-018-2924-7

**Published:** 2018-10-03

**Authors:** Shigeo Saito, Ying-Chu Lin, Yukio Nakamura, Richard Eckner, Kenly Wuputra, Kung-Kai Kuo, Chang-Shen Lin, Kazunari K. Yokoyama

**Affiliations:** 1 Saito Laboratory of Cell Technology, Yaita, Tochigi 329-1571 Japan; 2 College of Engineering, Nihon University, Koriyama, Fukushima 963-8642 Japan; 3 School of Dentistry, College of Dental Medicine, Kaohsiung Medical University, Kaohsiung, 807 Taiwan; 4 Cell Engineering Division, RIKEN BioResource Center, Tsukuba, Ibaraki 305-0074 Japan; 5 Department of Biochemistry and Molecular Biology, Rutgers, New Jersey Medical School-Rutgers, The State University of New Jersey, Newark, NJ 07101 USA; 6 Graduate Institute of Medicine, College of Medicine, Kaohsiung Medical University, Kaohsiung, 807 Taiwan; 7 Department of Surgery, Kaohsiung Medical University Hospital, Kaohsiung, 807 Taiwan; 8 Department of Biological Sciences, National Sun Yat-sen University, Kaohsiung, 804 Taiwan; 9 Faculty of Molecular Preventive Medicine, Graduate School of Medicine, The University of Tokyo, Tokyo, 113-0033 Japan

**Keywords:** Cancer stem cells, Epigenetics, Induced pluripotent stem cells, Organoid culture, Reactive oxygen species, Somatic cell nuclear transfer

## Abstract

The ability to control the transition from an undifferentiated stem cell to a specific cell fate is one of the key techniques that are required for the application of interventional technologies to regenerative medicine and the treatment of tumors and metastases and of neurodegenerative diseases. Reprogramming technologies, which include somatic cell nuclear transfer, induced pluripotent stem cells, and the direct reprogramming of specific cell lineages, have the potential to alter cell plasticity in translational medicine for cancer treatment. The characterization of cancer stem cells (CSCs), the identification of oncogene and tumor suppressor genes for CSCs, and the epigenetic study of CSCs and their microenvironments are important topics. This review summarizes the application of cell reprogramming technologies to cancer modeling and treatment and discusses possible obstacles, such as genetic and epigenetic alterations in cancer cells, as well as the strategies that can be used to overcome these obstacles to cancer research.

## Introduction

The use of human embryonic stem cells (ESCs) is a promising approach in the clinical applications of regenerative medicine and cancer research. However, the use of such ESC derivatives poses a major ethical dilemma, in that embryos need to be destroyed or compromised to produce ESCs. Therefore, more pragmatic alternatives, including reprogramming, are required to pave the path for the clinical application of pluripotent stem cells (PSCs) in humans. Currently, reprogramming technologies are divided into three approaches: (i) somatic cell nuclear transfer (SCNT) technology, (ii) induced PSC (iPSC) technology, and (iii) direct reprogramming (DR) technology [1].

### SCNT, iPSC, and DR technologies

SCNT technology generates totipotent cells using an enucleated oocyte injected with a nucleus isolated from differentiated somatic cells [2]. In mammals, the reprogramming capability of somatic cells to an undifferentiated state was first substantiated by the birth of cloned sheep [3]. In a rather different context, ESCs derived from the inner cell mass cells of blastocysts also exhibit pluripotency with indefinite cell division and the ability to differentiate to all three germ layers [4]. The invention of methods for the induction of human iPSCs derived from somatic cells opened a new era of research, as it allowed researchers to derive an almost infinite number of new iPSCs that can be used as a source for autologous cell-based therapy, disease modeling, drug screening, and biomedical engineering [5–13]. The current methodologies generally reprogram somatic cells to iPSCs via serial passages in the presence of reprogramming factors (OCT4, SOX2, KLF4, and c-MYC [OSKM], as well as NANOG and LIN28) under adherent culture conditions on a feeder layer or on extracellular matrix (ECM) components [14].

Reprogramming can also be induced by other methods using chemicals that promote the establishment of the core transcription circuitry of stem cells [15–18]. For example, an over 200-fold increase in reprogramming efficiency was reported for culture media supplemented with antagonists of transforming growth factor beta (TGF-β) signaling and mitogen-activated kinase/extracellular signal-regulated kinase (MEK–ERK) inhibitors, and by passaging the cells in the presence of thiazovivin, which is an inhibitor of the Rho-associated coiled-coil containing protein kinase (ROCK) [19].

A potentially important twist to reprogramming techniques has stemmed from the observation that pluripotency factors, such as OCT4 and LIN28, are markers of a group of stem cell-like cells in ovarian cancers [20]. Several studies have shown that the pluripotency factors used to generate iPSCs also exhibit tumorigenic capability, suggesting that reprogramming and cellular transformation might occur via overlapping pathways [21–28].

Therefore, reprogramming protocols involving the expression of oncogenic pluripotency factors might cause tumorigenesis by disrupting the epigenetic marks for the correct gene expression circuitry. For example, inhibition of the expression of the tumor suppressor gene encoding TP53 not only enhanced the reprogramming of fibroblasts into iPSCs [29], but also generated transformed CSCs from differentiated cells [30]. Moreover, it has been demonstrated that overexpression of c-MYC in immortalized mammary epithelial cells favored tumor formation via epigenetic cell reprogramming [31]. The authors provided evidence that this tumorigenesis was caused by epigenetic reprogramming, as the oncogenic enhancers were reactivated in the cancer cell counterparts. Furthermore, recent works have illustrated an important role of the epigenetic reprogramming of chromatin modifications in the evolution of cancer metastasis [32–34]. These articles emphasize the fact that reprogramming can lead to the formation of tumor-initiating cells that acquire stem cell-like phenotypes. Interestingly, the three-dimensional (3D) tumor sphere-forming assay is a unique model of cancer that can be used to investigate malignant heterogeneity in tumorigenesis [34, 35]. Therefore, the tumorigenic potential of the use of reprogrammed stem cells for clinical applications should be recognized and new approaches for safe stem cell therapy should be developed.

DR (or transdifferentiation) technology, which reprograms somatic cells to other differentiated lineages or multipotent stem cells or progenitors, has also been developed [36]. DR introduces target cell-specific, defined transcription factors into recipient somatic cells, which are reprogrammed to the target cells by bypassing the pluripotent stage during lineage conversion, thus possibly avoiding teratoma formation [37–39]. Human and mouse somatic cells have been converted into myoblasts, beta islet cells, neurons, and neural stem cells using DR technology [40–43]. DR is assumed to shorten the preparation period for cell replacement therapy and has the highest potential for clinical application. Nevertheless, to obtain the final target cells, this technique remains time consuming in practice. In addition, compared with SCNT and iPSC technologies, DR exhibits the lowest efficiency of successful reprogramming to PSCs [44, 45]. This problem needs to be overcome.

### Cancer stem cells (CSCs)

The CSC hypothesis was proposed over 140 years ago [46] and postulates that cancers arise from a rare subpopulation of cells that are endowed with both tumor and stem cell features. CSCs are resistant to drug and radiation therapies. It is believed that CSCs are self-renewing cancer cells that have clonal tumor-developing capability and clonal long-term repopulation ability [24, 47–49]. One model proposes that CSCs are derived from genetically and epigenetically altered stem or progenitor cells that reside in their original niches and acquire oncogenic growth advantages to sustain tumor mass. Thus, these CSCs might possess similar features to normal stem cells and are well adapted to the niche environments [50].

### Cancer cell reprogramming

An identical set of reprogramming factors (OSKM) can be delivered to cancer cells derived from almost all tissues to generate induced pluripotent cancer cells (iPCCs) [51–53]. Such iPCCs appear to have a CSC-like state after the reprogramming process [49, 53–55]. Alternatively, depending on the type of cancer, the introduction of a single gene (a process referred to as DR, see above) can be sufficient to activate multipotency and induce tumor formation. In normally unipotent basal or luminal mouse mammary epithelium, the induction of an activated gene encoding the phosphatidylinositol-4,5-biphosphate kinase catalytic subunit alpha (*PIK3CA*) was sufficient to trigger the reprogramming of these cells into a multipotent mammary epithelial stem cell-like state and to give rise to breast tumors that displayed a similar cellular heterogeneity to that of human breast cancers [55, 56]. Slightly elevated levels of c-MYC were sufficient to reprogram and dedifferentiate luminal mammary epithelial cells into a stem cell-like state, resulting in the widespread decommissioning of transcriptional enhancers associated with differentiation-specific genes and the reactivation of genes associated with multipotent breast epithelial stem cells [31]. The latter study also showed that c-MYC and an activated PIK3CA allele collaborate in inducing multipotency and in increasing the number of tumor-initiating cells. Schwitalla et al. [32] demonstrated that enhanced NF-kB signaling was able to activate WNT in intestinal cells and induced dedifferentiation of nonstem cells to acquire stem cell-like properties. Another report showed that the constitutively active SMAD2/3 can interact with other factors on OCT4 target loci and potentiate DR conversion with multiple types of transcription factors from myoblasts to adipocytes, B cells to macrophages, and fibroblasts to neurons. Thus, they might be the common cofactors that potentiate diverse cell fate conversions with master genes [57].

**Fig. 1:**
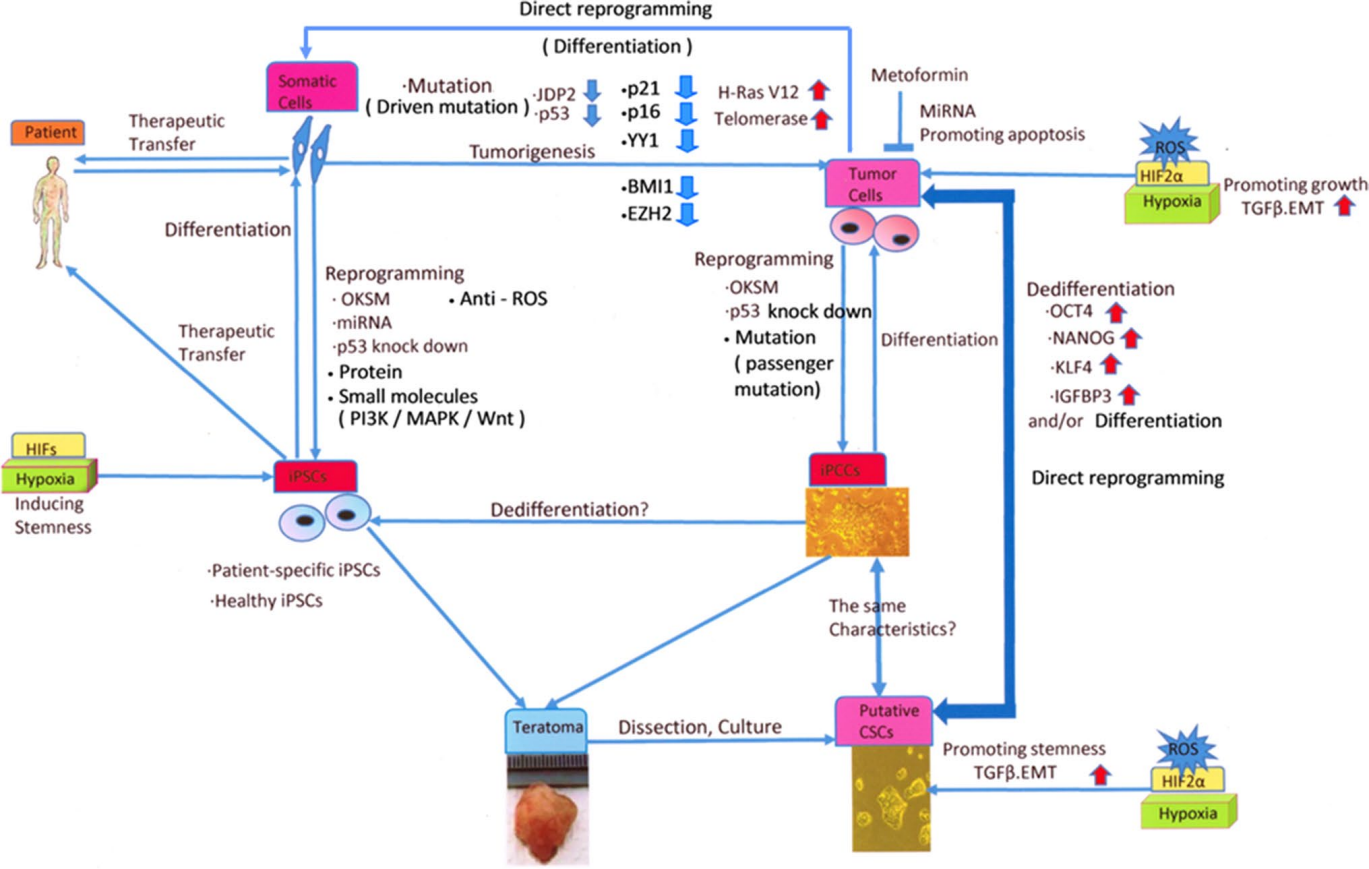
Schematic model of the interaction between reprogramming to pluripotency and tumorigenesis. The reprogramming of somatic cells to pluripotency is performed by overexpressing reprogramming factors (such as OCT4, KLF4, SOX2, c-MYC, NANOG, and miRNAs) and inhibiting tumor suppressor genes (such as those encoding p14^ARF^, p16^Ink4a^, p21^Cip1^, and p53), to reset their fate toward a state of pluripotency, which is a dedifferentiation process that resembles tumor development. Patient-specific or healthy iPSCs are used in cell-based therapy after inducing differentiation to appropriate types of cells, for transplantation into patients. For example, iPCCs were derived by introducing OSKM factors and knocking down vector shTP53 in tumor cells in a manner similar to that described in iPSC protocols. The teratomas that are formed after the transfer of iPCCs to SCID mice are then dissected out, and isolated cells can form putative CSC-like phenotypes. The various malignancy characteristics observed in iPCCs seem to depend on differences in tumor cell types. In contrast, CSCs can be derived by an OCT4-mediated dedifferentiation process in tumor progression, even in somatic cells, via the stable expression of telomerase, the H-Ras V12 mutant, and inhibition of the p53 and retinoblastoma protein (pRB) pathways. CSCs can also be derived directly from tumor cells via the overexpression of OCT4, NANOG, KLF4, and IGFBP3, in a dedifferentiating manner. Putative CSCs and iPCCs are expected to be used in studies of drug screening or cancer-initiation mechanisms in the field of human cancer therapeutics. Hypoxia enhances the reprogramming of somatic cells, and HIFs directly regulate the factors that are needed for self-renewal and multipotency in cancer cells and CSCs. Furthermore, hypoxia increases the production of ROS, which promote cell development and EMT in CSCs via the TGF-β signaling pathway and drive CSCs to produce VEGF, which induces angiogenesis

Accordingly, cancer cells that have been reprogrammed via the introduction of a single or several genes, which are capable of triggering a stem cell phenotype, can be a good model of several aspects of cancer research (Fig. 1), such as the study of cancer heterogeneity and niches, the elucidation of the mechanisms of cancer initiation and progression, epigenetic reprogramming, screening of compounds as therapeutic or re-differentiating agents, and induction of cell death/senescence for cancer ablation therapy [58, 59].

## Advantages provided for cancer research by cancer cell reprogramming

Heterogeneity of cancer cells in the same patient can arise for multiple reasons [58–60]. First, heterogeneity can be generated by stochastic genetic [61] or epigenetic changes [62]. Clonal evolution confers heritable differences among cancer cells. Second, heterogeneity can arise through the interaction between cancer cells and environmental alterations within the tumors [63]. Third, heterogeneity can be derived from a minor subpopulation of tumorigenic CSCs, which can generate diverse non-tumorigenic cells and constitute a tumor mass. In addition, tumorigenic CSCs can be transplanted between immune-deficient mice and reestablish phenotypic heterogeneity inside the newly formed tumor [64]. These sources of heterogeneity are not mutually exclusive [65]. The cellular heterogeneity encountered in many human tumors may represent a specific niche, which is an important contributing factor to the maintenance of tumor stem cells.

Genetically, some tumors, such as medulloblastomas [66], hepatocellular carcinomas [67], small cell lung carcinomas (SCLCs) [68], and pancreatic adenocarcinomas [69], are composed of such genetic subpopulations of each specific cancer [70]. Different cell subpopulations with a certain cell identity may be dominantly represented by cell reprogramming. For example, breast cancer cells with PIK3CA mutations may present as CSCs after MYC-induced reprogramming [31]. Thus, reprogramming technology might help identify heterologous subsets among cancer progenitor cells. Single-cell RNA sequencing technology has now been used to identify and quantitate these subtypes of cancer cells [71]. Such high-throughput single-cell RNA sequencing technologies have the potential to promote the understanding of cancer generation in the decomposition of heterogeneous cell populations and of the heterogeneity of cells associated with various tumorigenic stages. This technology allows the identification of the cellular subpopulations and the delineation of novel cell markers in the hematopoietic [71], respiratory [72], hepatobiliary [73], and pancreatic [74, 75] lineages, as well as in the intestine [76]. Recent progress in single-cell RNA sequencing led to the identification of the heterogeneous origins of CSCs in gliomas [77], breast cancers [78], myeloid leukemias [79], bladder cancers [80], and colorectal cancers [81]. These heterogeneities stem from the original unidentified subpopulations that arise during the step of induction of cancer-specific iPSCs. Henceforth, this single-cell sequencing technique can be applied to cancer-specific iPSCs. The limitations of this technique in the identification of heterogeneity are also presented. The major sources of genetic and phenotypic variations among iPSCs can be assessed by including gene copy numbers and the extent of epigenetic changes, indicating that this problem could be solved in the near future and that the real heterogeneity of such CSCs may be identified [82].

### Study of the microenvironmental niches of cancer stemness and organoid culture

Patient-specific iPSCs could be applied as cancer models for mechanistic studies and drug development, as well as for studying interactions with cancer niches. The newly developed 3D cell cultures of patient-derived iPSCs might be useful for understanding the roles of cellular microenvironments. Primary tumors at a late stage of cancer development represent differentiated cell lineages. The animal models that are available currently fail to provide ideal systems because of their genetic differences. The 3D and organoid cell culture of iPSCs could provide useful systems that are appropriate for modeling human cancers, which would be clinically important for drug screening and the development of therapies.

Stem cells grow in their own cellular microenvironments, termed stem cell niches. In these niches, both interactions between cells and the ECM and their diffusible signals are important for development. Niches have been used to identify mammalian stem cells in various epithelial tissues from normal samples and cancers [83]. The niches are composed of fibroblasts, immune cells, endothelial and vascular progenitor cells, or ECMs and network signals composed of cytokines and growth factors [79]. CSCs are also capable of forming niches representing tumor microenvironments [84]. During tumor progression to a more malignant stage, CSCs in the primary tumor depend on the tumor microenvironment or on the CSC niches that are located within it [85]. Thus, the reprogramming of cancer cells to generate iPCCs can provide critical information that can be used to understand the role of such microenvironments.

To elucidate the role of niches, the recently developed technique of organogenesis also provides useful information [86]. Recent techniques of organoid formation from brain, intestine, kidney, liver, lung, ovary, pancreas, and stomach cells provide basic knowledge on the cross-talk between CSCs and their microenvironment. This technique can be used in clinical applications, including cancer modeling, drug screening, microorganism infection, and therapy using new gene editing technologies, such as CRISPR/Cas9, to identify the critical genes, respectively. Thus, patient-derived organoids might be critical for future use in cancer research, for drug screening, and for mechanistic studies of CSCs and their microenvironments [87].

### Merits of the application of this technique to cancer modeling and cancer therapy

Cancer cell reprogramming can be used as a model to understand tumorigenesis and to develop regenerative therapies. In some cases, such reprogramming advances oncogenic capacity even further. Thus, after dedifferentiation, reprogrammed cancer cells exhibit a more severe cancer phenotype because of the genetic alterations or oncogenicity of the reprogramming factors that were used [40, 52, 88–90].

#### Leukemia

The reprogramming of the chronic leukemia KBM7 line into iPCCs using the transcription factors OSKM led to resistance to an inhibitor of the *Bcl*–*Abl* fusion oncogene in these cells, but not in the parental cells [52]. In another case, primary chronic myelogenous leukemia (CML)-derived iPCCs were shown to be resistant to imatinib. However, CML-iPCCs-derived hematopoietic cells recovered sensitivity to this drug. These findings indicate that the pathological features of the initial disease were recapitulated [88].

#### Gastrointestinal cancers

Nagai et al. [90] also reprogrammed gastrointestinal cancer cell (GCC) lines using OSKM. These iPCCs were sensitized to chemotherapeutic drugs and differentiation-inducing protocols at an early stage, but longer culture of these cells resulted in more aggressive features compared with the parental cells. Thus, the authors speculated that the cancer-specific iPCCs were prone to genetic instability via genetic or epigenetic alterations, including oncogenic *c*-*Myc* activation. Human pancreatic ductal adenocarcinoma (PDAC) cells were reprogrammed to generate iPCCs and injected into SCID mice. The reprogrammed cancer cells then produced the pancreatic intra-epithelial neoplastic lesions that can progress to invasive tumors [40]. Miyoshi et al. [53] used four different GCC lines to obtain iPSC-like cells. These GCC-iPSCs were generated by ectopic expression of OSKM and oncogenes, such as *BCL2* and *KRAS*, and short-hairpin RNAs (shRNAs) against the tumor suppressor genes, such as *TP53, p16*^*Ink4a*^*, PTEN, FHIT*, or *RB1.* These iPSC-like cells were more sensitive to 5-fluorouracil and drugs of differentiation–induction and exhibited reduced tumorigenicity in nonobese diabetic/severe combined immunodeficient mice. Kuo et al. [58] found that the positive feedback between *OCT4* and *c*-*JUN* increased with the onset of cancers. We hypothesized that the positive feedback regulation of OCT4 and c-JUN might promote the generation of liver CSCs.

#### Lung cancers

Mahalingam et al. [91] reprogrammed a non-small cell lung cancer (NSCLC) cell line using OSKM to generate NSCLC-iPCCs, which reversed the aberrantly dysregulated genes in cancer cells both epigenetically and transcriptionally, resulting in reduced oncogenicity in iPCCs.

#### Li‒Fraumeni syndrome (LFS)

LFS is a cancer hereditary syndrome caused by *TP53* germline mutations. Patients with LFS are susceptible to adrenocortical carcinoma, brain tumor, breast cancer, leukemia, osteosarcoma, and soft tissue sarcoma. LFS-patient-derived iPSCs have been generated [92]. LFS-iPSC-derived osteoblasts reproduced the hallmarks of osteosarcoma (OS), including defective osteoblastic differentiation and tumorigenicity. However, osteoblasts from LFS-derived iPSCs did not exhibit cytogenetic alterations in 18 regions that are usually associated with late-stage OS. The imprinting gene H19 was not upregulated in LFS osteoblasts during osteogenesis, and the restored forced expression of H19 in LFS osteoblasts improved osteoblastic differentiation and suppressed tumorigenicity. Thus, without differentiation, iPSCs were able to maintain stemness with higher expression of the H19 gene product, even though the *TP53* gene was mutated.

LFS-derived iPSCs provide several advantages compared with other models of LFS, such as (i) an unlimited supply of cells, (ii) a human platform, and (iii) access to the heterogeneity across cell types. Thus, LFS-derived iPSCs can provide great value in drug screening and testing in vitro. LFS-derived iPSC models enable the understanding of precise genome editing, three-dimensional (3D) organoid-based culturing systems, and subsequent organ-on-chip systems, which might facilitate anticancer drug discovery and provide a sophisticated model of cancer treatment [92].

### Merits of the development of therapeutics

A cell line of the blast crisis stage of CML was reprogrammed to generate CML-iPSCs [52]. CML was generated by mutating the *BCR–ABL* fusion gene, which caused enhanced cell expansion [93], while CML-iPSCs retained their differentiation potential. Thus, the maintenance of stemness and oncogenic expansion is a critical issue during differentiation. In a blast crisis, cells lose their ability to differentiate, and immature leukemia cells can overgrow instead. In the case of in vivo differentiation in teratomas, CML-iPSCs differentiate into all three germ layers, including hematopoietic cell lineages expressing CD34, CD43, and CD45. Cells with loss of the CML phenotype and independence from BCR–ABL signaling were resistant to imatinib. Differentiation of the cells into hematopoietic lineages in vitro rendered them sensitive to imatinib, suggesting the recovery of oncogenic dependency, as the CML-iPSCs underwent hematopoietic differentiation.

Kumano et al. [88] demonstrated that iPSCs derived from the primary tumors of two patients with CML exhibited stemness and differentiation to hematopoietic progenitors that expressed BCR–ABL. These iPSCs were prepared from imatinib-sensitive patients, but the iPSCs finally showed resistance to this drug and resembled CML stem cells after reprogramming. These cell lines might provide a good model system for understanding the mechanism of drug resistance and the role of stem cells in CML.

iPSCs might be useful for the development of personalized approaches to cancer treatment, as they would enable the discovery of a wide range of therapeutic agents against the genetic differences between individuals, which might aid the discovery of those that are ideal for each patient [94]. The identification of an efficient strategy to eliminate CSCs is a critical issue in cancer therapy. As CSCs are rare, iPSC technologies could be used to generate a large quantity of CSCs for subsequent applications [95, 96]. Nishi et al. [97, 98] generated mammary CSC-like cells that were used to screen compounds that selectively targeted CSCs, including salinomycin and withaferin A. Choi et al. [99] generated iPSC-derived hepatic cells from patients with α-1 antitrypsin (AAT) deficiency, to screen the Johns Hopkins Drug Library (3131 clinical compounds). Of the 262 compounds that led to decreased AAT accumulation by > 50%, 43 showed no side effects. Finally, the authors identified five hits that consistently decreased AAT levels in four AAT-deficient iPSC lines. Patient-derived iPSCs are also useful for the study of drug absorption, distribution, metabolism, excretion, and toxicity. Thus, the use of iPSCs is beneficial for the identification of CSC-related genes and for mechanistic studies of cancer induction, promotion, and progression.

### Study of metabolic shifts

Cancer cell reprogramming has the advantages of reconstituting cancer initiation and progression, which renders it an ideal model to investigate changes in cancer characteristics, such as metabolism, epithelial–mesenchymal transition (EMT)/mesenchymal–epithelial transition (MET), and metastasis.

The Warburg effect, via which cancer cells use glycolysis rather than oxidative phosphorylation in mitochondria for producing energy, is well known [100, 101]. Aerobic glycolysis, which is mediated by uncoupling proteins that uncouple oxidative phosphorylation from glycolysis [102–105], is enhanced in ovarian and breast cancers and when PSC pluripotency is induced. Lu et al. [106] generated iPSCs from patients with ataxia telangiectasia (AT) syndrome that mimicked the AT phenotype, including deregulated AT-mutated (ATM)-associated pathways and altered gene expression patterns in the pentose phosphate and mitochondrial oxidative phosphorylation pathways. Metabolic reprogramming of pyruvate utilization is a therapeutic target for the development of new reagents for cancer prevention [107], such as those affecting the inhibition of pyruvate dehydrogenase kinase [108]. The anti-hyperglycemic agent metformin is an interesting substance with therapeutic effectiveness. Although the action of metformin has not been explained fully, it is useful for the metabolic reprogramming of cancer cells [109]. Metformin promoted growth arrest in pancreatic tumor cells via direct impairment of fatty acid synthesis [110]. The antitumor effects of metformin appear to be correlated with microRNA (miRNA) modulation and increased expression of the AMP-activated protein kinase, leading to the modulation of targets that restore energy homeostasis by inhibiting hepatic gluconeogenesis [109].

### Analysis of EMT/MET

EMT/MET play critical roles during normal development, as they contribute to the formation of the mesoderm during gastrulation, as well as at subsequent stages of the development of neural crests and lung formation [111]. They are also hallmark of cancer initiation and metastasis. For example, EMT/MET inducers, such as SNAIL1/2 or TWIST1/2, are associated with relapse and survival in several cancers, such as those that arise in mammary, colorectal, and ovarian tissues, suggesting that EMT/MET pathways are associated with poor outcomes of cancer patients [112, 113]. The expression of EMT/MET genes is correlated with cancer progression in colon cancers, papillary thyroid carcinomas, and breast carcinomas [113], and in the development of metastases in melanomas [114]. In xenotransplantation assays, iPSCs derived from human sarcoma cell lines proliferated more slowly than did their parental counterparts and exhibited necrosis and lower expression of EMT markers [115]. During reprogramming, initial methylation followed by demethylation of the promoters of 32 oncogenes and 82 tumor suppressor genes were demonstrated, showing that pluripotency factors can suppress the features of cancer phenotypes, restore differentiation potentials, perturb epigenetics via DNA methylation, and alter cancer-related gene expression.

### Molecular approach to the study of cancer metastasis

Compared with their normal counterparts, cancer cells exhibit widespread alterations in DNA methylation patterns and an altered organization of open and condensed chromatin because of profound changes in epigenetic chromatin marks [116, 117]. Additional epigenomic reorganization takes place during tumor progression to metastasis [118, 119]. Each metastatic event establishes a new tumor nodule and is, thus, by definition, carried out by CSCs [120]. Recent studies have begun to shed light on the molecular mechanisms that lead to metastasis. Not surprisingly, the changes appear to be tumor-type specific. For example, during SCLC progression to metastasis, the expression of the transcription factor nuclear factor 1b (Nfib) increases by several fold, in part from the amplification of the *Nfib* gene, resulting in the activation of new distal regulatory elements (i.e., transcriptional enhancers) and the implementation of a neuroendocrine transcriptional program that drives metastasis [121]. In PDAC, the genomes of primary tumors and their metastases are largely similar, suggesting that epigenetic reprogramming might be the primary force driving the transition [122]. Two different reports have described widespread chromatin and gene-enhancer reprogramming during PDAC progression [33]. Those authors investigated matched PDAC cells from the same patients from either proximal (peritoneum) or distant (lung and liver) metastatic sites. PDAC metastases from distant sites were dependent on the oxidative pentose phosphate pathway for the maintenance of their malignant gene expression programs. Roe et al. [34] also used a mouse PDAC model and found that the transition to a metastatic state was accompanied by massive FoxA1-driven enhancer activation. The newly activated genes rendered cells more invasive, and they assumed a cell fate resembling that of the embryonic foregut endoderm.

These examples suggest that the reprogrammed cancer cells displayed various cancer phenotypes that provided a prevention technology and insights into cancer biology and the progression of cancers.

## Obstacles to cancer cell reprogramming

This reprogramming technique for cancer cells remains immature; therefore, additional trials are needed to understand the weakness that exists currently in cell reprogramming for the translational research of cancers.

### Mutations in the genome

Usually, cancers are produced by “driver” mutations at the initiation stage and, subsequently, by positive selection and clonal expansion, which lead to the accumulation of “passenger” mutations [123, 124]. The “driver” mutations confer an advantage to the proliferation and development of cancers. In contrast, the “passenger” mutations do not affect the fitness of cancer clones significantly [125–127]. Recent advances in deep genomic sequencing technologies have led to the identification of these mutations in some oncogenes and tumor suppressor genes, which are the hallmark drivers of certain cancers [128]. However, whether these genetic mutations become a barrier to cancer cell reprogramming remains unclear.

In addition, many studies have demonstrated that the process of cell reprogramming may cause genomic alterations, such as chromosomal aberrations, copy number variations (CNVs), and single-nucleotide variations. For example, trisomy 12 is an aberration that is observed commonly in ESCs and iPSCs [72–75]. Some cell cycle-related genes and NANOG are located on chromosome 12; thus, trisomy 12 might result in alterations in proliferation and reprogramming [76, 77]. The amplification of chromosomes 8 and X, as well as of other chromosomes, was also detected in iPSCs [72, 73]. iPSCs may acquire CNVs during reprogramming or from the mosaicism that is present in the parental cells; however, CNVs are lost gradually by cell passaging, with selective pressure for the deletion of tumor suppressor genes in early cell passages and duplication of oncogenes at a later time [75, 82–84, 129]. Single-nucleotide mutants in iPSCs are identified by high-throughput next-generation sequencing analyses. These analyses have identified an average of ten protein-coding mutations per human iPSC line [68, 85]. Thus, further investigation is required to identify approaches aimed at preventing these mutations during the cellular reprogramming of cancer cells.

The use of young donor cells is one possible way to overcome this issue, because mutations in mitochondrial DNA increase with age in human iPSCs [88, 130]. If the preparation of autologous donor cells is difficult, human histocompatibility antigen (HLA)-matched allogenic cells can be used to replace them in reprogramming, to generate iPSCs. It might not be necessary to prepare autologous donor cells, because human HLA-matched umbilical cord blood-derived iPSCs, which do not show a higher rate of point mutations, are useful sources of allogenic iPSC-based cell therapies [131]. Yamanaka’s group and the RIKEN Cell Bank in Japan are initiating this project to cover most Japanese HLAs to produce allogenic iPSCs with lower mutation rates that could be used as iPSCs bank stocks.

### Epigenetic alterations

The process of fibroblast reprogramming using Yamanaka’s factors (OSKM) includes three steps: initiation, maturation, and stabilization [132]. The initiation step is characterized by the expression of genes that encode proteins involved in MET via the silencing of SNAIL1/2, suppression of TGF-β signaling, and upregulation of CDH1 [133]. In the maturation step, the expression of exogenous 4Fs is repressed and pluripotent-related genes, such as *NANOG, SALL4,* and *ESRRB*, are expressed in their stead. In the stabilization step, other pluripotent marker genes are expressed for full reprogramming.

In the initiation step, cells undergoing reprogramming exhibit downregulation of the H3K79me2 epigenetic markers located around MET-related genes. A decreased H3K79me2 level indicates the inhibition of mesenchymal properties through transcriptional repression. Subsequently, the genes encoding poly-(ADP-ribose) polymerase-1 and the ten–eleven translocation (TET) family 2 (TET2) are recruited to the *NANOG* and *ESRRB* loci, which direct the transition from the initiation to the maturation phase [134]. In the maturation and stabilization phases, epigenetic silencing of the exogenous genes and enhancing of chromatin remodeling represent the resetting of epigenetic modifications in these reprogramming-related genes [135].

TET-mediated DNA demethylation at CpG islands (at the *ESRRB* and *OCT4* loci via an interaction with NANOG) promotes gene expression and helps maintain the pluripotency of stem cells [136]. In cancer cells, high levels of DNA methyltransferase 1 (DNMT1) and DNA methyltransferase 3A/3B (DNMT3A/3B), as well as suppression of TET, have been detected [137, 138]. The repressed function of TET in cancer cells might impair pluripotency and genomic reprogramming. The epigenetic features of cancer cells, such as high expression of DNMTs, low expression of TETs, and overexpression of histone deacetylases (HDACs), might be an obstacle to the reprogramming process.

**Fig. 2:**
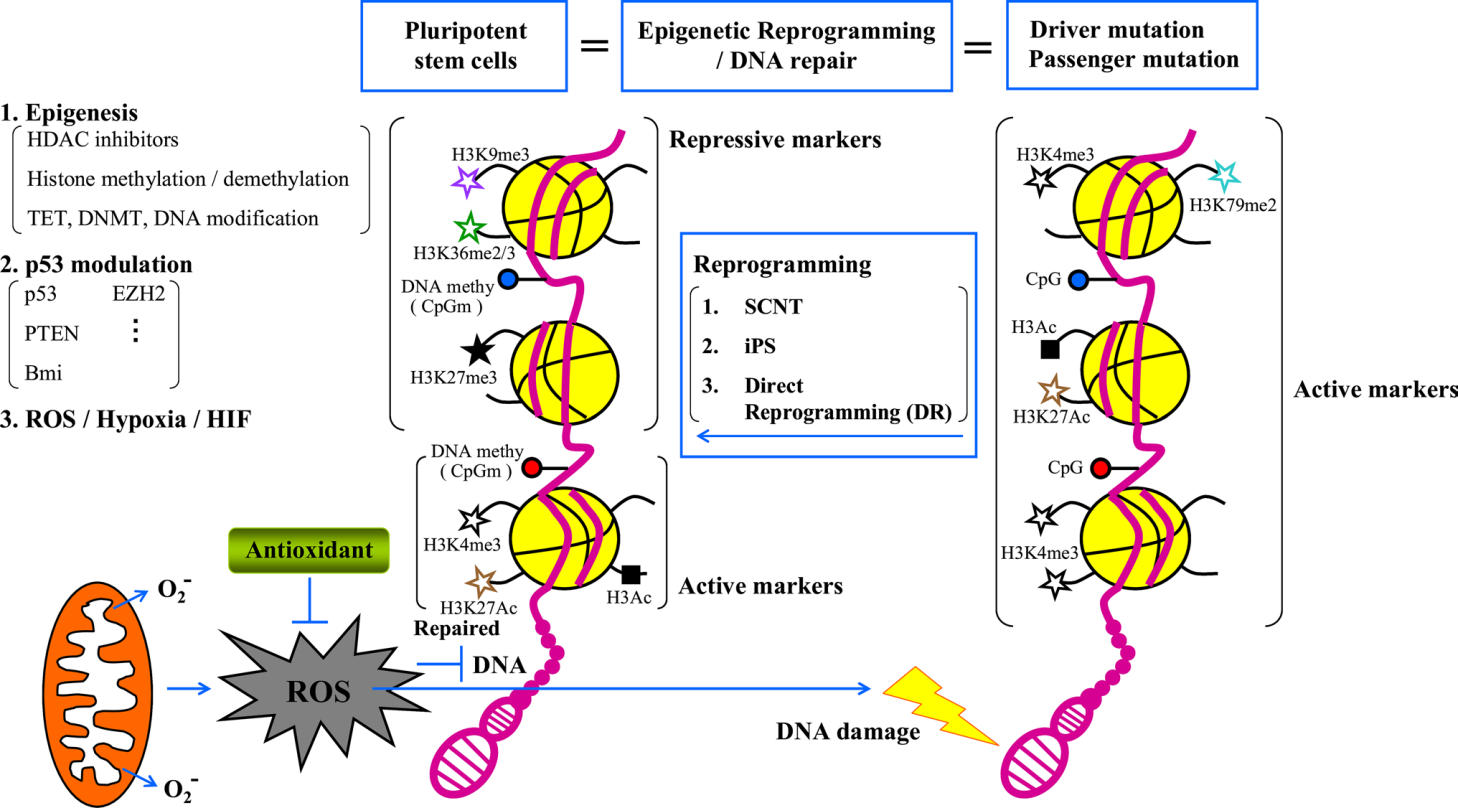
Schematic model of the mechanisms via which epigenesis, p53, and ROS‒hypoxia‒HIFs promote reprogramming efficiently and genome integrity in PSCs. Cancer cells with driver and passenger mutations might be overcome by epigenetic reprogramming and DNA repair to induce the formation of PSCs with correct plasticity. Active chromatin with active histone markers (H3K4me3, H3K79me2, H3Ac, and H3K27Ac) should be repressed by repressive markers (H3K9me3, H3K36me2/3, and H3K27me3) at specific regions by three different reprogramming methods (SCNT, iPSC, and DR). Forced expression of reprogramming factors increases the levels of ROS that are generated in mitochondria, which in turn causes DNA damage and undermines both reprogramming efficiency and the genomic integrity of iPSCs. Antioxidants can promote reprogramming efficiency and safeguard the stability of the genomes of iPSCs by inhibiting ROS production and exerting non-antioxidant functions, including modulating epigenetic modifiers, and histones

In addition to DNA methylation, histone modifications also play critical roles in cancer cell reprogramming (Fig. 2). Histone marks, such as H3K27me3, H3K9me3, H3K4me3, and H3K27ac, are targets for the reprogramming of cancer cells. The catalytic subunit of the polycomb repressive complex 2 (PRC2) enhancer of zesta homolog2 (EZH2) mediated transcriptional repression by introducing H3K27me3 [139]. In breast cancers, B-cell lymphomas, and prostate cancer, EZH2-mediated H3K27me3 permitted the silencing of tumor suppressor genes [140–143]. Accordingly, in myeloid malignancies, loss of EZH2 function was sufficient to induce a self-renewal-supporting transcriptional program and leukemogenesis. These reports indicate that the deregulation of the H3K27me3 landscape—hence, the transcriptional repression—is the driving force behind the emergence of CSCs, independent of the original EZH2 mutation [144–147]. The mixed lineage leukemia (MLL) histone methyltransferase is also involved in histone modification. MLL requires the repressive activity of the polycomb repression complex 1 (PRC1), which monoubiquitinates histone H2A at lysine 119 (H2AK119Ub1) or trimethylates histone H3 at lysine 4 (H3K4me3), and then cooperates with PRC2 to mediate transcriptional repression [139]. The Bmi1 subunit of PRC1 mediates the repression of tumor suppressors in myeloid progenitors [148, 149] and is required for the inhibition of tumor suppressor genes that is necessary to initiate the self-renewal of CSCs in solid tumors [150]. The control afforded by ATP-dependent chromatin remodeling complexes, such as SWI/SNF, ISWI, CHD, and INO080, represents another pathway of epigenetic regulation in mammals [151]. The genes encoding the SWI/SNF complex are mutated in > 20% of human cancers. Loss of SMARCB1, a subunit of the SWI/SNF complex, drives malignant rhabdoid tumors and is associated with the blocking of differentiation, reprogramming toward an oncogenic transcriptional program, and activation of cancer signaling [152, 153]. ARID1A, another subunit of SWI/SNF, is a tumor suppressor in colon cancers and its loss activates an oncogenic program and promotes the development of invasive colon adenocarcinomas in the mouse [154]. Taken together, these findings show that deregulations of the DNA methylation and histone modification landscapes represent key steps in the onset of the generation of CSCs.

In general, it might be better to define precise chromatin regulatory regions, including physical constraints such as the insulators and topologically associated domains, while lamina-associated domains are mainly localized along large organized chromatin modifications, as well as with the heterochromatic regions of silenced genes in cells [155]. Alterations in these higher order structures have been linked to the control of tumorigenesis [156]. Transcriptional enhancers are enriched for the binding of chromatin factors such as p300/CBP, a major histone acetyltransferase that mediates the formation of H3K27AC, and its mediator, a long-range interaction facilitator [157]. The correct ordering and functional integrity of these modifiers with transcription factors and enhancers should be clarified in terms of the generation and expansion of CSCs [158].

However, recent development in high-throughput tools that allow the examination of chromatin structure, such as DNase I-, formaldehyde-assisted isolation of regulatory elements- (FAIRE-), and assay for transposase-accessible chromatin (ATAC) sequencing, can be used to extend our knowledge of epigenetic regulation during cell reprogramming [159, 160]. Conversely, the incorporation of hyperdynamic histone variants at enhancers (H2A.Z and H3.3) might render the chromatin less stable and facilitate the initial access to transcription factors [161–163], demonstrating the presence of oncogenic enhancers that are involved in cancer commitments. However, many questions remain unanswered: how can cancer reprogramming erase the epigenetic memory of stem or differentiated cells? How can oncogenic enhancers be maintained? Which molecules are involved in the initiation and progression of cancer genotypes with expanding CSCs of each tumor type [164]? CRISPR-mediated epigenome editing may be a promising technique to identify the key *cis*-elements in the genomes of CSCs [165–168]. The origins of cancer stemness and the manners in which stemness genes and oncogenes might be separated remain unclear. However, these breakthroughs and the identification of new drugs targeting epigenetic processes (epi-drugs) may open a new era of therapeutic strategies to target CSCs for reprogramming.

## Potential key factors to overcome the obstacles to cancer cell reprogramming

Several reprogramming enhancers are thought to be able to overcome the problems raised above. These reprogramming enhancers can be divided into the following categories: modulators of tumor suppressor proteins, hypoxia and reactive oxygen species (ROS), and cellular signaling and chromatin modifiers (Table 1, Fig. 2).

**Table 1:** List of modulating factors for enhancing the efficacy of reprogramming

Technology	Modulators	Function	Type	References
SCNT	Serum starvation	Cell cycle	Medium supplement	[134]
	TSA, VPA, Scriptaid	Epigenesis	Medium supplement	[215–217]
	FBS	Proliferation	Medium supplement	[218]
	Vitamin C	ROS	Medium supplement	[219, 220]
	Hypoxia	ROS	Medium supplement	[221]
	5-Azacytidine	Epigenesis	Medium supplement	[222–224]
	KDM4A	Epigenesis	Gene	[45]
	H1foo	Epigenesis	Gene	[225]
iPSCs or iPCCs	TSA, VPA, thiazovivin, chemicals	Epigenesis	Medium supplement	[15–19, 226–228]
	5-Azacytidine	Epigenesis	Medium supplement	[226]
	SB431542	TGF-β inhibitor	Medium supplement	[229]
	Vitamin C	ROS	Medium supplement	[230]
	FBS	Proliferation	Medium supplement	[175]
	Serum starvation	Cell cycle	Medium supplement	[231]
	Inhibition of DOT 1L	Epigenesis	Gene	[2]
	AID	Epigenesis	Gene	[232, 233]
	Overexpression of MYC	Epigenesis	Gene	[31]
	Activation of PIK3CA, Smad2/3	Epigenesis	Gene	[55–57]
	Inhibitors of p53 or PTEN	Proliferation	Gene	[29, 51, 169–177]
	Inhibitors of Brigent/Arid3A	Proliferation	Gene	[234]
	Inhibitors of cyclin D1	Cell cycle	Gene	[177]
	Overexpression of E-Cad	Mesenchymal–epithelial transition	Gene	[235]
	Hypoxia	Proliferation	Other	[236]
	Pattern	Epigenesis	Other	[237]
	Overexpression of YY1/Sox2, OCT4/Bmi1	Proliferation	Gene	[94]
	YAPI/TAZ	Proliferation	Gene	[238]
	TERT–EZH2	Proliferation/chromatin	Gene	[239]
Direct reprogramming	Hypoxia	ROS	Other	[44]
	SB431542	TGF-β signal	Medium supplement	[197]
	Inhibitor of p53	Proliferation	Gene	[112, 240]
	Inhibitor of Bmi1	Epigenesis	Gene	[199]
	Overexpression of HMGA2	Epigenesis	Gene	[43]
	miR-125a/HK2	Metabolism	Gene	[241]
	SoxB1, SoxE, SoxF	Stemness	Gene	[200]
	C-Myc, Klf4, Sox9	Pluripotency	Gene (mouse dermal fibroblasts to chondrogenic cells [iChon])	[242]
	Sox, EZH2	Epigenesis	Gene (mouse fibroblasts to iNSCs)	[202]
	NF-κB, LEF-1	Signal	Gene (human fibroblasts to sweat gland-like cells)	[243]
	ASCL1, ISL1, NEUROD1, BRN2, HB9, LHX3, HYT1L, NGN2	Pluripotency	Gene (human fibroblasts to motor neuron)	[203]
	JMJD3	Epigenesis	Gene (bone marrow progenitor to liver cells)	[204]
	Ascl1, Zfp238, Sox8, Dlx3	Pluripotency	Gene (mouse fibroblasts to iN)	[201]
	GATA4, HAND2, MEF2C, TBX5(AGHMT), ZNF281	Pluripotency	Gene (human fibroblasts cardiomyocytes)	[198]

This table is a modified version of the one published by Kwon et al. [244]

### Tumor suppressor proteins

Transient inhibition of the gene encoding the tumor suppressor protein 53 (TP53) or the phosphatase and tensin homolog protein (PTEN) increases reprogramming efficiency [51, 169–175]. During the transient inhibition of tumor suppressors, cell proliferation is increased, and cell cycle arrest, apoptosis, and senescence are inhibited, which are favorable conditions for reprogramming. For example, the introduction of a dominant-negative *TP53* [176] or *shRNA*–*TP53* [59] into cells increased the efficiency of reprogramming. However, cyclin D1 was reported to be an obstacle to reprogramming to a pluripotent state [177]. Inhibition of TP53 was also effective in the direct conversion of human fibroblasts to dopaminergic neurons [178].

### Hypoxia and ROS scavenger JDP2

Hypoxia induces the expression of hypoxia-inducible factors (HIFs). Two main HIFs, HIF1α and HIF2α, are essential for the metabolic changes that are required to generate iPSCs, whereas HIF2α is detrimental at the late stage of reprogramming of human cells. Prolonged HIF2α stabilization represses reprogramming because the tumor necrosis factor (TNF)-related apoptosis-inducing ligand and apoptosis are induced. Hypoxia treatment used during the induction of iPCCs might induce an increase in tumorigenicity, indicating the possibility that the targets of HIFs might be enhancers of CSC genes [89]. Moreover, hypoxia and expression of HIFs are required for the survival of CSCs [47, 179] and trigger ROS-dependent EMT [180]. Both hypoxia and elevated levels of glycolysis are conducive to the maintenance of stem cell features. It has been proposed that hypoxic culture conditions and reduced mitochondrial respiratory activity might increase the generation of iPSCs and inhibit the differentiation of ESCs [181, 182]. For example, it has been shown that hypoxia increases the DR efficiency of somatic cells into induced neural stem cells (iNSCs) or induced cardiomyocytes (iCMs) [183].

ROS are toxic oxygen derivatives and radicals derived from aerobic metabolism that lead to cellular damage and cell death [184, 185]. Increased levels of ROS reduce cell viability and decrease the reprogramming efficiency. In contrast, ROS scavengers lower oxidative stress, thereby increasing reprogramming efficiency. The reprogramming efficiency is significantly increased by adding vitamin C to the cell reprogramming culture medium [186]. The c-Jun dimerization protein 2 (JDP2) was identified as a cofactor that enhances antioxidant response activity [187, 188]. JDP2 acts as a repressor protein that inhibits cell proliferation; it induces cellular senescence during tumor development and participates in ROS homeostasis to inhibit cell damage by ROS [188]. These molecular features of JDP2 are also controlled by hypoxia and HIFs. Oxidative stress also induces angiogenesis via increased expression of angiogenetic marker genes, such as the vascular endothelial growth factor (VEGF) gene [189]; moreover, hypoxia stimulates the production of VEGF by CSCs [190]. Taken together, these findings suggest that the stemness of CSCs might be affected by extrinsic factors, such as hypoxia, ROS, and signaling between CSCs and environmental niches (e.g., TGF-β and the tumor necrosis factor-α, WNT, NOTCH, SHH signals and ECM stiffness, and some CSC-related transcription factors) [180, 190–193].

### Signaling modulators and chromatin modifiers

In addition to the reprogramming factors mentioned above, other reprogramming enhancers, including miRNAs and lncRNAs [194], have been emerging. These are also factors that are key to overcoming the obstacles to cancer cell reprogramming (Table 1). Kaufhold et al. [195] found that Yin Yang 1 (YY1) was a transcriptional repressor for stemness factors such as *BMI1, SOX2*, and *OCT4*. YY1 contributes to enhancer‒promoter interactions in a manner that is analogous to the DNA interaction mediated by CTCF [196]. The existence of a regulatory loop between the nuclear factor kappa b (NF-kB)–PI3K–AKT pathway and downstream products, such as BMI1, OCT4, SOX2, and YY1, has also been noted. Thus, modulation of YY1 and NF-kB–PI3K–AKT signaling may contribute to cell reprogramming. TGF-β pathway inhibitors, such as SB431542, increased the efficiency of the reprogramming of adult cardiac fibroblasts to iCMs [197]. Moreover, the B-lymphoma Mo-MLV insertion region 1 homolog (Bmi1) is a barrier to cardiac reprogramming. The inhibition of Bmi1 leads to an increase in the level of the active histone mark H3K4me3, and to a decrease in the level of the repressive mark H2AK119ub at cardiogenic loci, resulting in cardiac gene expression and increased reprogramming efficiency [198]. The zinc finger protein 281 (ZNF281) also enhances the direct conversion of fibroblasts to iCMs [199]. Because the high-mobility group AT-hook 2 (HMGA2) is involved in higher order chromatin compaction, its overexpression might help relax the nucleosome into a more open state for DR. Among the known reprogramming factors, the SOX family members, especially those of the SOXB, SOXE, and SOXF subclasses, are potent drivers of direct somatic cell reprogramming into multiple lineages [200].

Chromatin modifiers, such as the EZH2 and ASCL1 components, are also useful for DR to iNSCs or motor neurons [183, 201–203]. JMJD3 has been reported as an epigenetic enhancer of lineage conversion from bone marrow progenitors to liver cells [204]. Agathocleous et al. [205] and Cimmino et al. [206] reported that vitamin C regulated HSCs and suppressed leukemogenesis by modulating TET2 activity. Vitamin C is a cofactor of Fe^2+^- and alpha-ketoglutarate-dependent dioxygenases. Vitamin C modulates stem cell function, potentiates the reprogramming of fibroblasts to iPSCs, and inhibits the aberrant self-renewal of HSCs by enhancing the activity of Jumonji-1C domain-containing histone demethylases or TET DNA hydroxylases. Thus, vitamin C restores TET function in HSCs and might represent an adjuvant agent for treating leukemia and other cancers [207]. Vitamin C treatment has been applied to cancer cells such as melanomas [208], in which it increased 5-hydroxymethylcytosine content and resulted in the inhibition of tumor cell invasion and clonogenic growth in soft agar. Moreover, vitamin C is also useful for the metabolic reprogramming of cancer cells [207]. In addition to these identified reprogramming enhancers/modulators, further studies are still required to verify and overcome the problems of epigenetic reprogramming, mutations, and ROS-metabolic reprogramming in mitochondria and the endoplasmic reticulum.

## Conclusions and future perspectives

The basic techniques of cell reprogramming have their own merits for each cell type. Both the SCNT and iPSC technologies have the potential to erase genetic and epigenetic modifications in cancers and return the cells back to their stemness phenotype. Although DR- and classical iPSC-based reprogramming have considerable potential, their low efficiency of successful reprogramming and poor reproducibility limit the development of research in this field. Several obstacles must be overcome in the use of cell reprogramming. It will be challenging to maintain homeostasis, regulate ROS production, and maintain normal aging in the directly reprogrammed and pluripotent cells. Reprogramming enhancers are possible modulators of cancer and their microenvironments (niches) that might allow the application of this technology to translational research. Among the tumor suppressor genes, the status of TP53 signaling in CSCs plays a critical role in maintaining the stemness and expansion of cancer cells [49, 209]. To study therapeutic models in cancer research, 3D organoid models of ductal pancreatic cancers have provided a new spectrum of models of tumor progression by forming neoplasms that proceed to form invasive and metastatic carcinomas [210]. The organoid methodology is a useful system that can be used to identify the characteristics of malignancy, and the creation of complete tissues or neoplastic cancer organoids in vitro might provide better models of cancers in the future [35, 211–214]. Moreover, the CRISPR/Cas9 approach is believed to be a new breakthrough technology that can be used to correct cancer genomes for clinical applications [214]. However, Haapaniemi et al. [212] and Ihry et al. [213] demonstrated that CRISPR/Cas9 genome editing technology induces p53-mediated DNA damage and that, in human PSCs, p53 inhibits CRISPR/Cas9-induced genome editing. Thus, the tumor suppressor product of TP53 remains critical for overcoming this problem. Efforts to harness the versatility of iPSCs to model human cancers and to screen for effective therapeutics will undoubtedly accelerate translational cancer research from the laboratory to the bedside.
